# *Drosophila* Importin-α2 Is Involved in Synapse, Axon and Muscle Development

**DOI:** 10.1371/journal.pone.0015223

**Published:** 2010-12-06

**Authors:** Timothy J. Mosca, Thomas L. Schwarz

**Affiliations:** 1 F. M. Kirby Neurobiology Center, Children's Hospital Boston, Boston, Massachusetts, United States of America; 2 Department of Neurobiology, Harvard Medical School, Boston, Massachusetts, United States of America; Columbia University, United States of America

## Abstract

Nuclear import is required for communication between the cytoplasm and the nucleus and to enact lasting changes in gene transcription following stimuli. Binding to an Importin-α molecule in the cytoplasm is often required to mediate nuclear entry of a signaling protein. As multiple isoforms of Importin-α exist, some may be responsible for the entry of distinct cargoes rather than general nuclear import. Indeed, in neuronal systems, Importin-α isoforms can mediate very specific processes such as axonal tiling and communication of an injury signal. To study nuclear import during development, we examined the expression and function of Importin-α2 in *Drosophila melanogaster*. We found that Importin-α2 was expressed in the nervous system where it was required for normal active zone density at the NMJ and axonal commissure formation in the central nervous system. Other aspects of synaptic morphology at the NMJ and the localization of other synaptic markers appeared normal in *importin-α2* mutants. Importin-α2 also functioned in development of the body wall musculature. Mutants in *importin-α2* exhibited errors in muscle patterning and organization that could be alleviated by restoring muscle expression of Importin-α2. Thus, Importin-α2 is needed for some processes in the development of both the nervous system and the larval musculature.

## Introduction

To ensure a proper long-term response to stimuli, cells must have communication between the cytoplasm and the nucleus. Signals arising from diverse pathways must be imported into the nucleus where they can affect gene transcription. This process is largely accomplished through the function of importin proteins [1], which mediate the active import of protein cargoes through the nuclear pore complex [2].

The importin family comprises two major classes of protein: Importin-α and Importin-β [3]. In many cases, these proteins form a ternary complex with a cargo molecule [4]: Importin-α mediates cargo binding through a nuclear localization signal (NLS) while Importin-β binds Importin-α and mediates translocation through the nuclear pore [5]. In this model, Importin-α confers cargo specificity to the import machinery [6] and is the necessary intermediary between the cargo and Importin-β. Neurons have adapted this mechanism to connect the synapse to the nucleus of the cell. The cellular underpinning of learning and long-term memory is thought to depend on proper synapse-to-nucleus communication through the importins [7], [8]. Indeed, the involvement of Importin-α in a diverse array of specific neuronal processes has been shown [9], [10], [11], [12], [13]. Therefore, elucidating the roles of different Importin-α homologues can illuminate both the general mechanisms of nuclear import and the specific contributions of individual importins to neuronal function.

There are three evolutionary clades of Importin-α homologues and each is singly represented in the *Drosophila* genome [4], [14]. All three homologues are required for proper development of male and female germline tissue [15], [16], [17], [18], [19], [20]. In addition, they function outside of the germline: *importin-α1* is involved in wing patterning [18], *importin-α2* may be involved in cell proliferation and cell cycle progression [21], [22] and *importin-α3* is involved in cell fate decisions [23], heat stress response [24] and antagonism of Wnt signaling [25]. Importin-α3 function has also been examined in the nervous system: mutations in *importin-α3* fail to import a synaptic dSmad2 signal into the nucleus and show defects in proper axonal tiling of photoreceptors [11]. Further, only Importin-α3 is absolutely required for viability: germline clones fail to develop embryos and mutations die at the first- to second-instar transition while *importin-α1* and *importin-α2* mutants survive through to adulthood [15], [16], [25]. Their survival suggests specific but not essential roles for *importin-α1* and *importin-α2*.

We recently identified a role for Importin-α2 outside the germline. Importin-α2 contributes to postsynaptic development of the neuromuscular junction (NMJ) by permitting the import of a Fz2-derived signal, the C-terminal peptide of the Fz2 receptor, into muscle nuclei [26]. As Importin-α2 is expressed during larval development and in developing neuroblasts [21], [22], it is likely to be involved in additional aspects of neuronal development. Here, we show that Importin-α2 is expressed throughout the larval nervous system and is involved in determining both the density of active zones at the larval neuromuscular junction and axon connectivity in the central nervous system (CNS). We also find that *importin-α2* is required in the muscle for normal patterning and organization of the larval body-wall musculature.

## Materials and Methods

### *Drosophila* Stocks

*Drosophila* strains were raised at 25°C on cornmeal-molasses food. The isogenic stock *y, w; FRT42D* was used as the wild-type control. The following alleles and transgenic strains were used: *imp-α2^D3^*, *imp-α2^D14^* [II, 20]; *Df(3L) α1S1*, referred to here as *Imp-α1^Df^* [III, 18], 24B-GAL4 [III, 27]; Elav-GAL4 [III, 28]; UASt-Importin-α2 [III, 15].

### Immunohistochemistry

Larvae were grown on standard grape juice agar plates at low density at 25°C. Wandering third-instar larvae were dissected and processed for immunohistochemistry as described [26] with the following primary antibodies: mouse anti-Brp (NC-82) 1∶250 [29], mouse anti-CSP (6D6) 1∶100 [30], mouse anti-Fasciclin II (1D4) 1∶20 [31], mouse anti-Fasciclin III (7G10) 1∶100 [32], rabbit anti-GluRIIC 1∶2500 [33], rabbit anti-Importin-α2 1∶100 [19], mouse anti-Repo (8D12) 1∶100 [34], rabbit anti-Synaptotagmin I 1∶4000 [35]. FITC-, Cy3- or Cy5-conjugated secondary antibodies were used at 1∶200 (Jackson ImmunoResearch, West Grove, PA). Nerves were stained with antibodies to HRP at 1∶100 (Jackson ImmunoResearch, West Grove, PA) and muscles with Texas Red-conjugated phalloidin at 1∶300 (Invitrogen, Carlsbad, CA). Larvae were mounted in Vectashield (Vector Laboratories, Burlingame, CA) stored at −20°C until imaging.

### Imaging and Analysis Parameters

Larvae were imaged using an LSM 510 Meta laser scanning confocal microscope (Carl Zeiss, Oberkochen, Germany) and either a 63× 1.4 NA or 40× 1.0 NA objective. Images were processed in separate channels using the LSM software or Adobe Photoshop CS2 (Adobe Systems, San Jose, CA).

Scoring for bouton counts, commissural defects and muscle patterning errors were performed using a Nikon (Tokyo, Japan) E800 fluorescent microscope. For boutons, NMJs on muscles 6 and 7 of segments A2 and A3 on both the right and left sides were analyzed; bouton number was normalized to muscle surface area. Commissures were scored as defective if they had discontinuous axons at the midline of the ventral nerve cord. Muscle patterning was analyzed in segments A2 through A7. Genotypes were processed in the same tube and imaged with identical settings. Bruchpilot (Brp) and GluRIIC puncta were manually counted at MN4b synapses on muscle 4 from confocal z-stacks and terminal area was calculated using the threshold function in Metamorph for the HRP channel as previously [36].

Fluorescent intensity was measured in ImageJ as previously [26]: confocal z-stacks of the entire NMJ at muscle 4 were converted to multi-channel composite images. An ROI was drawn based on the anti-HRP channel and the mean fluorescence intensity measured in the other channels. Because the average anti-HRP fluorescence did not differ significantly between genotypes, direct comparison of the experimental labeling was possible.

Statistical analysis was conducted using Prism 5 software (Graphpad Software, La Jolla, CA) and significance (relative to wild-type unless otherwise noted) was calculated using a one-way analysis of variance (ANOVA) with a Dunnett post-hoc test to a control sample. When only two samples were tested, an unpaired student's t-test was used. Values are given as mean ± SEM; sample size (*n*) is described either in the figure legend or in the Results section.

## Results

### *Importin-α2* is Expressed in the Larval Nervous System

**Figure 1:**
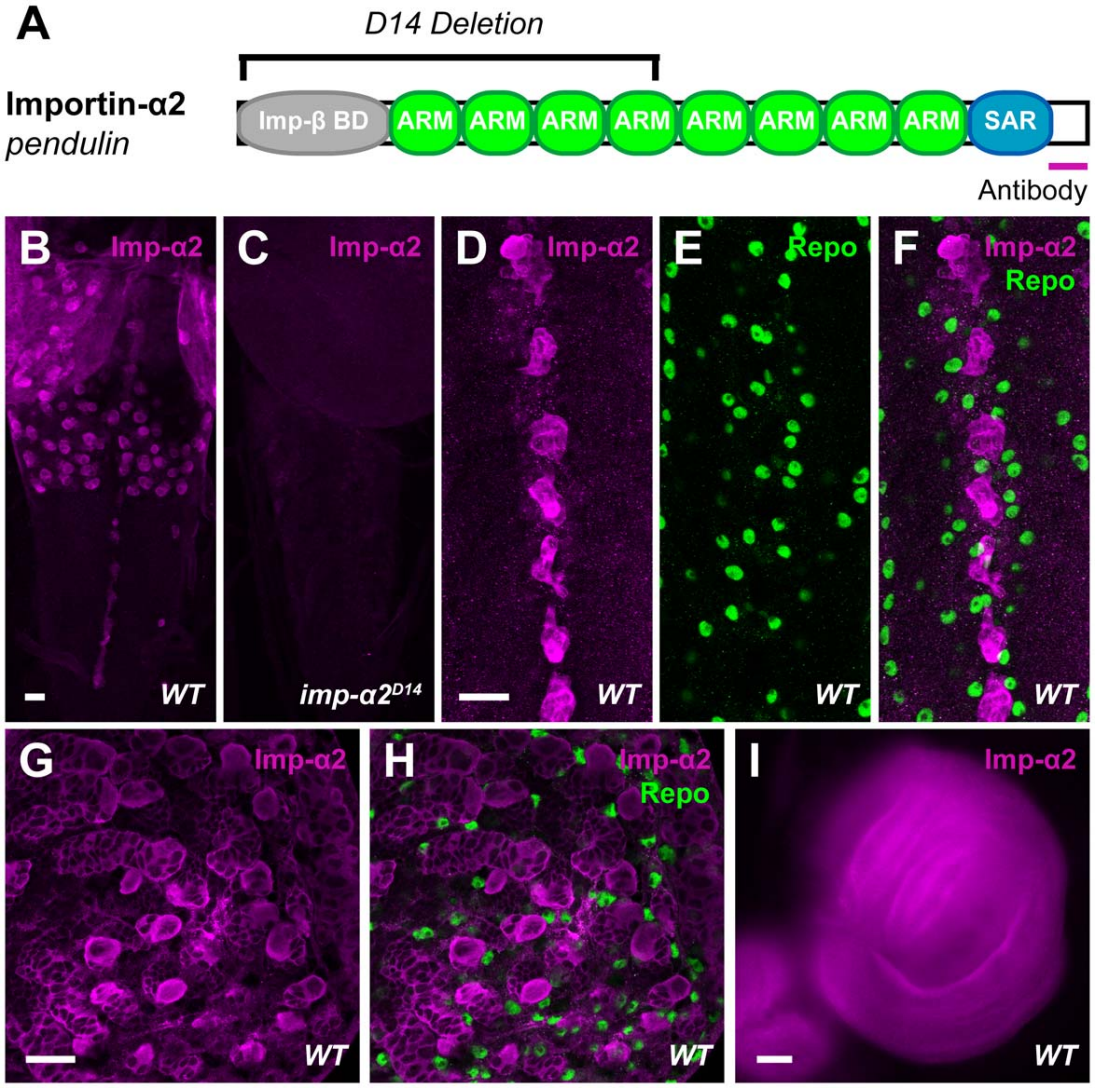
Importin-α2 is Expressed in the Larval Nervous System. (A) Diagram of the Importin-α2 protein reflecting the Importin-β binding domain (grey), eight Armadillo (ARM) repeats (green) and C-terminal SAR domain (blue). The extent of the D14 deletion [20] and the region against which polyclonal antibodies were raised [19] are indicated. (B,C) Representative confocal z-stacks of the ventral nerve cord (VNC) from wild-type (*y,w; FRT42D; +; +*) and *importin-a2* mutant (*y,w; imp-α2^D14^; +; +*) third-instar larvae stained with antibodies to Importin-α2 (magenta). The antibody labels a population of wild-type cell bodies within the brain lobes and at the midline of the VNC. (D–F) High magnification images of the midline of the VNC in wild-type larvae stained with antibodies to Importin-α2 (magenta) and the glial marker Repo (green). Repo does not overlap with Importin-α2 staining. (G–H) In high magnification images of the brain lobes of wild-type larvae, Importin-α2 (magenta) and Repo (green) immunoreactivities do not overlap. (I) A larval imaginal disc in a wild-type larva stained with antibodies to Importin-α2 (magenta). In all panels, scale bar = 10 µm.

*Drosophila* Importin-α2 is a 522 amino acid protein encoded by the gene *pendulin* [21] and comprises an N-terminal Importin-β binding domain and a series of ARM repeats followed by a SAR domain (Figure 1A) [37], [38]. To examine the function and expression of Importin-α2 in *Drosophila*, we used the deletion *imp-α2^D14^* [20], which removes the first half of the coding sequence (Figure 1A) and an antibody raised against the C-terminus of Importin-α2 [19].

Importin-α2 expression has previously been demonstrated during embryonic development [15], [19], [20], including in neuroblasts [21], [22], and in larval muscle nuclei [26]. In third-instar ventral nerve cords (VNC), we observed that anti-Importin-α2 recognized cells in the brain lobes and at the midline of the VNC (Figure 1B). This staining was absent in the *imp-α2^D14^* deletion mutant (Figure 1C). The Importin-α2-immunoreactive cells of the nerve cord midline (Figure 1D–F) and brain lobes (Figure 1G–H) were not labeled by antibodies to the glial marker Repo. Importin-α2 is therefore likely to be abundant in a subset of neurons. Additional neuronal populations may contain Importin-α2 at levels below the threshold for detection. Further, and consistent with previous *in situ* data [22], we observed robust expression of Importin-α2 in larval imaginal discs (Figure 1I).

### Active Zone Density at the NMJ Is Controlled by Neuronal *Importin-α2*

**Figure 2:**
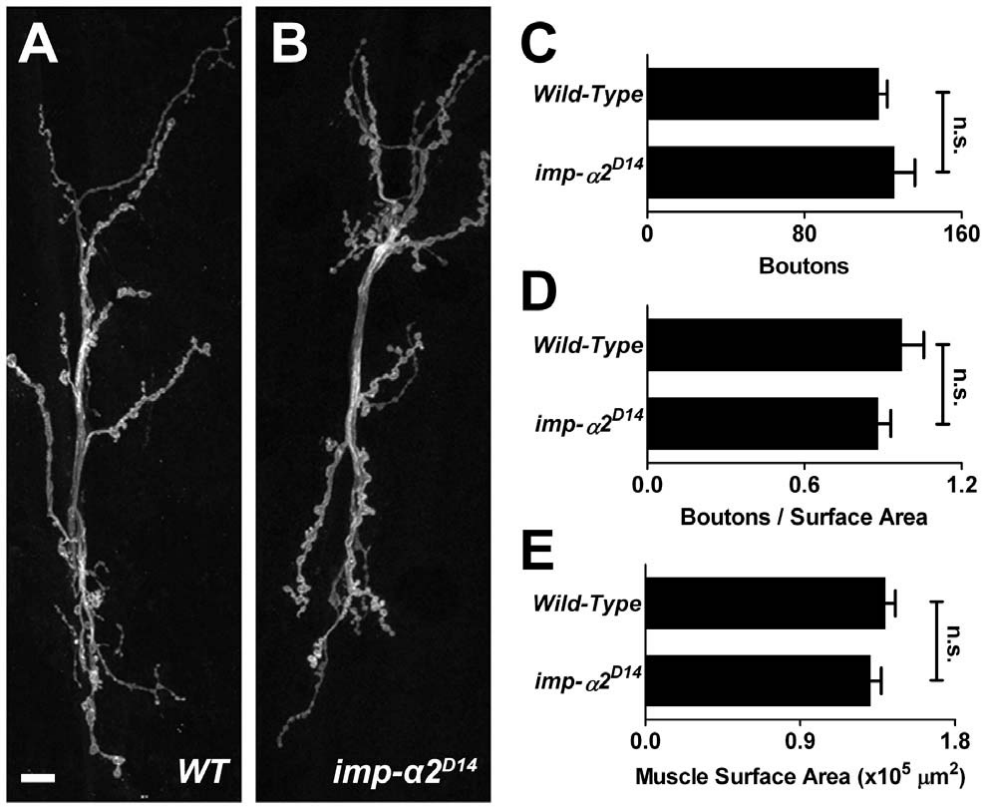
Normal Bouton Number in *importin-α2* **Mutants.** (A–B) Representative confocal images of third-instar larval NMJs from wild-type (*y,w; FRT42D; +; +*) and *importin-α2* mutants (*y,w; imp-α2^D14^; +; +*) stained with antibodies to HRP. Scale bar = 10 µm. (C–E) Quantification of bouton number (C), bouton number normalized to muscle surface area (D) and muscle surface area (E) in *wild-type* and *importin-α2* larvae. No significant differences were detected. In all cases, *p*>0.2 and error bars represent S.E.M. For *wild-type*: *n* = 29 NMJs, 15 larvae and for *importin-α2*: *n* = 22 NMJs, 12 larvae.

As we observed expression of Importin-α2 in the nervous system and muscle [26], we examined the NMJ for anatomical phenotypes. *importin-α2* mutant NMJs appeared grossly similar to wild-type controls (Figure 2A–B) and possessed similar numbers of boutons (Figure 2C). The surface area of the muscle was also unchanged (Figure 2E) and thus the matching of bouton count to muscle size was unaltered (Figure 2D).

**Figure 3:**
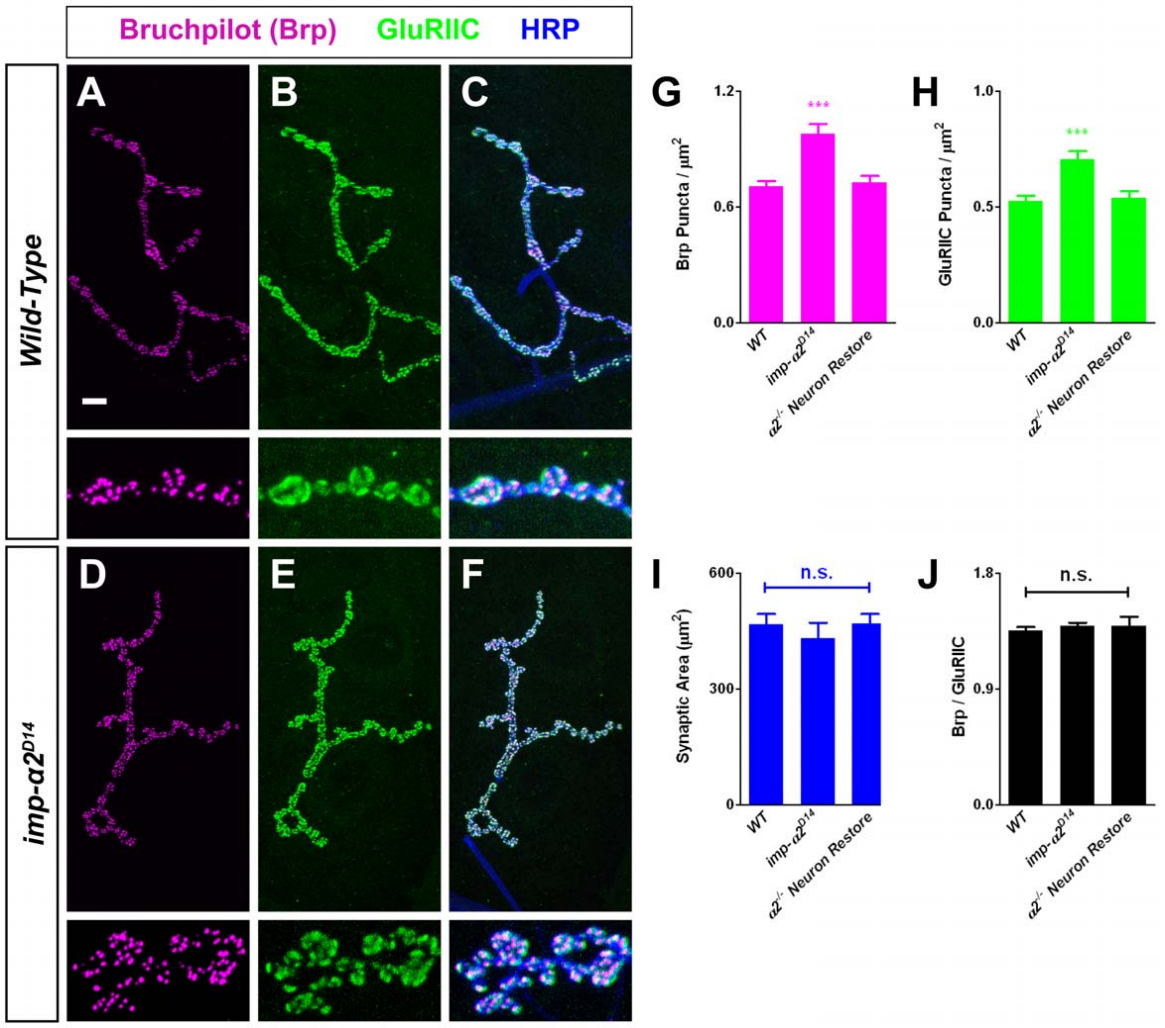
Loss of Neuronal Importin-α2 Increases Active Zone Density. (A–C) Representative confocal images of wild-type (*y,w; FRT42D; +; +*) larval NMJs from muscle 4 stained with antibodies against Bruchpilot (magenta), GluRIIC (green) and HRP (blue). Insets show high magnification images of individual boutons. Each Brp punctum is apposed by a corresponding GluRIIC punctum. (D–F) Representative confocal images stained as above in *importin-α2* mutants (*y,w; imp-α2^D14^; +; +*). No defects in apposition are observed, but the density of both Brp and GluRIIC puncta is increased. Scale bar = 10 µm for full NMJ, 4 µm for high magnification images. (G–H) Quantification of Brp and GluRIIC puncta in wild-type larvae, *importin-α2* mutants, and *importin-α2* mutants with restored neuronal expression of Importin-α2 (*y,w; imp-α2^D14^; elav-GAL4/UASt-Importin-α2; +*). In *importin-α2* mutants, the density of Brp and GluRIIC puncta per µm^2^ is increased by 40% and restored to wild-type levels by neuronal restoration of Importin-α2 expression. *** *p*<0.0001. (I–J) The synaptic area (as determined by anti-HRP staining of terminals) and the ratio of Brp to GluRIIC puncta are unchanged in the mutants. Error bars represent S.E.M. For all cases, *n*≥2 NMJs from 6 larvae.

We also examined the localization and expression of synaptic markers in wild-type and *importin-α2* mutant larvae. We labeled NMJs with the monoclonal antibody NC-82, which recognizes the active zone component Bruchpilot [29], [39] and with antibodies against GluRIIC, an essential subunit of the glutamate receptor complex [33]. In wild-type, each presynaptic Brp punctum is apposed by a postsynaptic GluRIIC punctum (Figure 3A–C). This alignment is essential for reliable synaptic transmission [33] and can be regulated by diverse signaling pathways [40], [41], [42]. In *importin-α2* mutants, we observed proper localization of both Brp and GluRIIC to the NMJ (Figure 3D–F) and no change in the total area of the NMJ as determined by anti-HRP immunoreactivity (Figure 3I). However, the number of puncta was increased by 39% for Brp and by 35% for GluRIIC compared to wild-type (Figure 3G–J). The number of active zone and glutamate receptor puncta thus increased in tandem and no examples were seen of active zones that lacked apposite receptor clusters. In contrast, *importin-α1* null larvae showed normal active zone densities (Figure S1). *importin-α3* mutants were not examined because they die at the first larval instar [16].

The phenotype indicated a requirement for nuclear import in controlling the density of active zones at the NMJ. To determine the origin of this phenotype, we expressed a UAS-Importin-α2 transgene [15] in all postmitotic neurons using the elav-GAL4 driver [28] in an *importin-α2* mutant background. In these animals, the densities of Brp and GluRIIC puncta (Figure 3G–I) were restored to wild-type levels Therefore, a neuronal pool of Importin-α2 is required for normal active zone density at the NMJ.

### Many Presynaptic Markers are Normal in *importin-α2* Mutants

**Figure 4:**
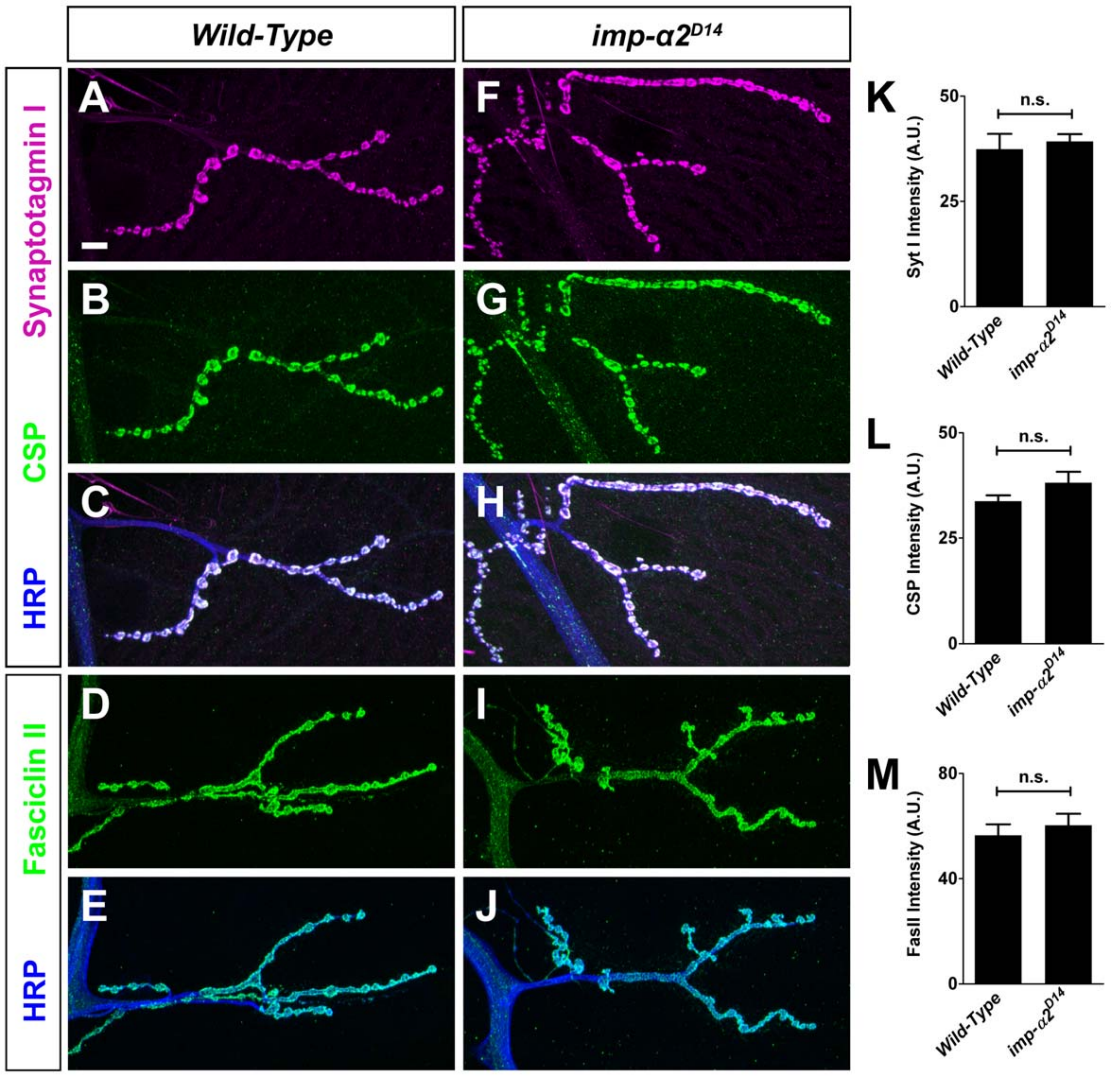
Common Synaptic Markers are Normal in *importin-α2* Mutants. (A–E) Representative confocal projections of the larval NMJ at muscle 4 stained with antibodies to Synaptotagmin I, Cysteine String Protein (CSP), Fasciclin II, and HRP, as indicated, in wild-type (*y,w; FRT42D; +; +*) larvae. (F–J) Representative confocal projections of *importin-α2* mutant larvae (*y,w; imp-α2^D14^; +; +*) stained as in (A–E). As in wild-type larvae, synaptic vesicle proteins and Fas II properly localize at the NMJ in *importin-α2* mutants. Scale bar = 10 µm. (K–M) Quantification of the intensity of immunoreactivity for Syt I (K), CSP (L) and Fas II (M) in wild-type and *importin-α2* mutant larvae. No statistically significant differences were observed. In all cases, *p*>0.4, *n* = 6 animals, 12 NMJs and the error bars indicate S.E.M.

As the loss of *importin-α2* resulted in an increase of Brp and GluRIIC puncta, we examined whether other synaptic proteins were altered. CSP and Syt I immunoreactivities, which mark synaptic vesicle populations [30], [43], were properly localized at *importin-α2* NMJs (Figure 4A–C, F–H) and the fluorescence intensity of each antibody signal was equivalent at wild-type and mutant NMJs (Figure 4K–L). We also examined Fasciclin II, a cell adhesion molecule necessary for proper synaptic structure and stabilization at the NMJ [44], [45]. Fasciclin II was correctly localized at the *importin-α2* NMJ (Figure 4D–E, I–J) and the fluorescence intensity of the synaptic Fas II signal showed no significant difference between wild-type and *importin-α2* larvae (Figure 4M). Therefore, while Brp and GluRIIC are altered at *importin-α2* mutant synapses, the localization and concentration of other synaptic markers are unaffected at these synapses.

### Aberrant Axonal Commissures in *Importin-α2* Larvae

**Figure 5:**
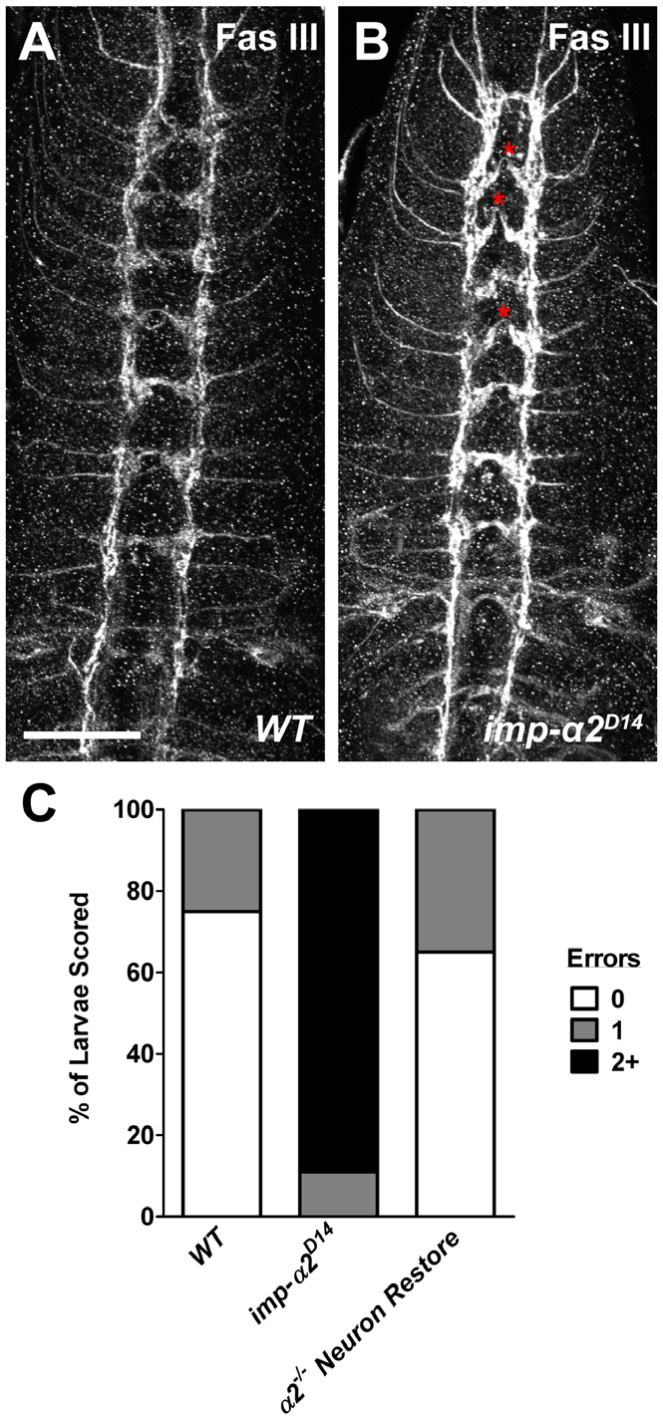
Axon Commissure Defects at the Midline of *importin-α2* Mutants. (A–B) Representative confocal projections of the ventral nerve cord midline in both wild-type (*y,w; FRT42D; +; +*) and *importin-α2* mutant (*y,w; imp-α2^D14^; +; +*) third-instar larvae stained with antibodies to Fasciclin III. In wild-type larvae, Fas III antibodies stained the central-most longitudinal tracts and commissural axons crossing the midline. While both the longitudinal tracts and commissures were visible in *importin-α2* mutants, there were frequent discontinuities in the staining of the commissures (red asterisks). Scale bar = 50 µm. (C) Quantification of larvae possessing no errors, single abnormal commissures or multiple abnormalities in wild-type controls, *importin-α2* mutant larvae, and *importin-α2* mutant larvae where neuronal expression has been restored (*y,w; imp-α2^D14^; elav-GAL4/UASt-Importin-α2; +*). For each genotype, *n*≥20 animals.

To examine the organization of the VNC, we stained wild-type and *importin-α2* mutants for Fasciclin III, a cell adhesion molecule [32] that recognizes crossing RP motor neuron axons [46]. In wild-type larvae, antibodies to Fas III stain one of the lateral axon tracts that run lengthwise through the nerve cord as well as a band of axons that cross the midline in each segment (Figure 5A). In *importin-α2* mutants, Fas III immunoreactivity was present on both the lateral axon tracts and crossing axons. However, the midline crossings were frequently discontinuous (Figure 5B, asterisks), appearing broken at the center. Whereas 75% of wild-type larvae had no apparent discontinuities of these commissural tracts and the remaining 25% had only a single discontinuity (Figure 5C), all *importin-α2* larvae had at least one such defect and 89% had 2 or more (Figure 5C). A maximum of 5 discontinuities were observed in a single mutant animal. The phenotype could be rescued by restoring expression of Importin-α2 in neurons using the elav-GAL4 driver: 67% of these animals had no axon tract errors while 33% had one defect. Thus, development of proper axon commissures in the CNS requires a neuronal function of Importin-α2.

### *importin-α2* Mutant Larvae Display Defects in Muscle Patterning

**Figure 6:**
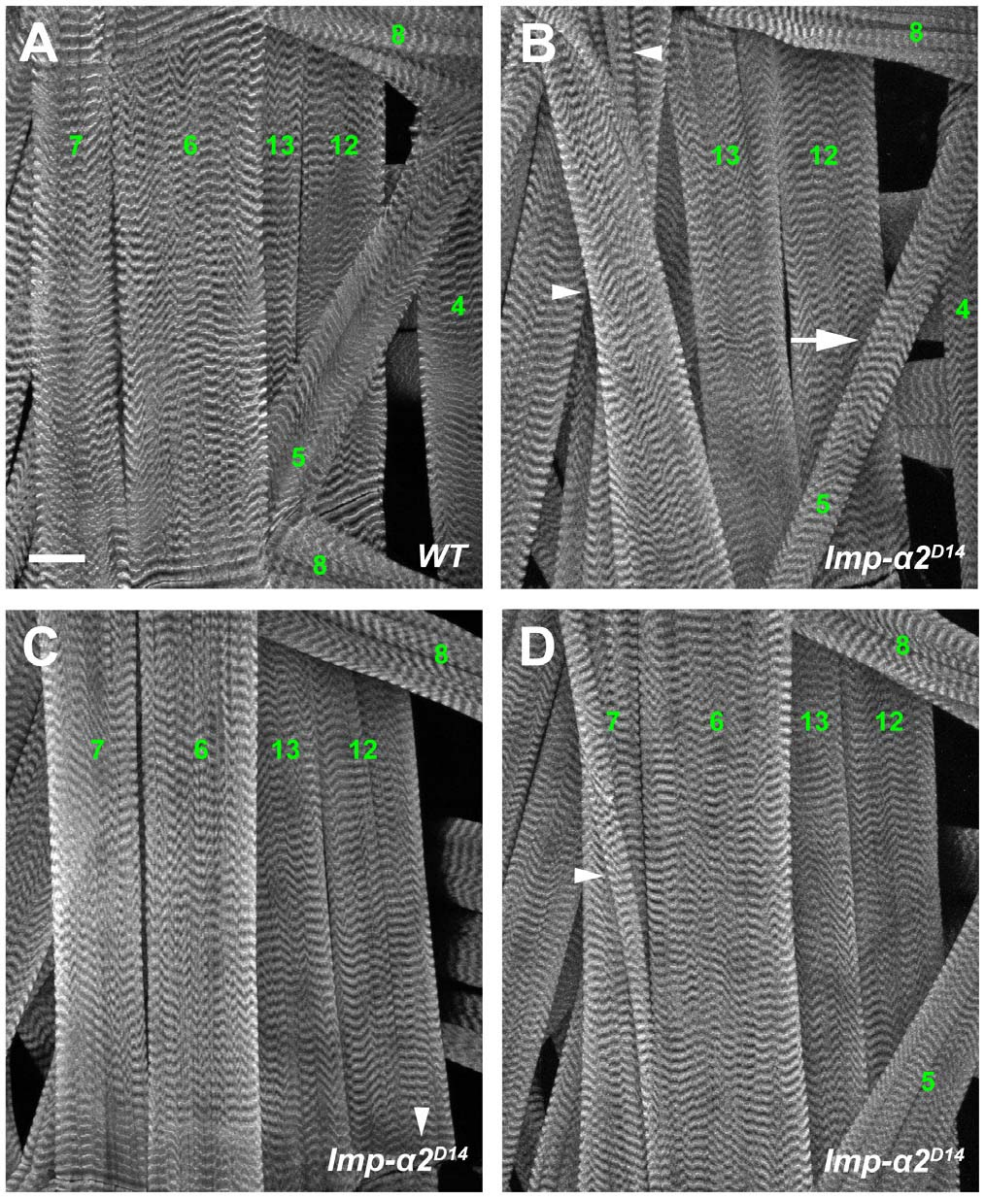
Muscle Patterning Defects in *importin-α2* Mutants. (A) Representative confocal image of a wild-type (*y,w; FRT42D; +; +*) larval muscle field stained with Texas Red-conjugated phalloidin. Muscles are labeled according to Crossley [47]. This represents a single hemisegment between the midline (at left of image) and muscle 4. Muscle 8 from the next anterior hemisegment is also visible. (B) An *importin-α2* mutant (*y,w; imp-α2^D14^; +; +*) with incorrect positioning of muscles 6 and 7 (arrowheads). In wild-type larvae (A), these muscles are parallel but they cross in some *importin-α2* mutants. Also, an abnormally thin muscle 5 is evident in this example (arrow). (C) Representative example of an *importin-α2* mutant where muscle 5 is absent but other muscles are normal. (D) Representative example of an *importin-α2* mutant in which an abnormally branched fiber has formed (arrowhead). In this case, the branch derives from muscle 6. These four categories of defect were observed in *importin-α2* mutants. Scale bar = 50 µm.

Importin-α2 is present in muscle nuclei and is required for normal postsynaptic development by mediating a synapse-to-nucleus Fz2 signal [26]. Larvae mutant for *importin-α2*, however, also have defects in the patterning of the larval body-wall muscles. Wild-type larvae possess a nearly invariant muscle pattern with the ventral longitudinal muscles 7, 6, 12 and 13 lying closest to and running parallel with the midline [47] while muscle 5 lies obliquely to these (Figure 6A). In *importin-α2* mutants, several types of errors were evident in the organization of the muscle fibers (Table 1). Most frequently, we observed a defect in muscles 6 and 7 whereby instead of running parallel, the two crossed, each appearing to insert improperly at the insertion site of the other (Figure 6B). Additionally, we observed two types of defect in muscle 5: either the muscle was abnormally thin (Figure 6B, arrow) or absent altogether (Figure 6C). We also observed instances of branched muscle fibers (Figure 6D). Unlike the other defects that were characteristic of a particular muscle or pair of muscles, the branching of fibers was observed at several positions. Although the muscle defects were quantified in the homozygous *imp-α2^D14^* larvae, they were also observed in other allelic combinations of *importin-α2* mutants (data not shown). In aggregate, although no deviations from the normal pattern were observed in 96 wild-type segments examined, a quarter of the 228 mutant hemisegments contained defects and every *importin-α2* mutant larva had at least 1 defective hemisegment (Table 1). Expressing Importin-α2 in muscles with the 24B-GAL4 driver largely reversed the patterning defects, but neuronal expression via elav-GAL4 did not (Table 1). The propensity to error in muscle patterning thus implies a function of Importin-α2 in muscle cells prior to the previously described requirement for this importin in the growth of the subsynaptic membranes [26].

**Table 1:** Frequency of Muscle Patterning Errors in *importin-α2* Mutant Larvae.

Genotype	*Wild-Type*	*imp-α2^D14^*	*imp-α2^D14^; elav-GAL4/UAS-Imp-α2 (Neuron Restore)*	*imp-α2^D14^; 24B-GAL4/UAS-Imp-α2 (Muscle Restore)*
Attachment Errors	0 (0%)	29 (13%)	8 (8%)	2 (2%)
Missing Muscles	0 (0%)	18 (8%)	6 (6%)	0 (0%)
Branched Muscle	0 (0%)	8 (4%)	20 (19%)	2 (2%)
Thin Muscles	0 (0%)	5 (2%)	6 (6%)	0 (0%)
Total Errors	0 (0%)	60 (26%)	40 (38%)	4 (4%)
Total Hemisegments	96	228	105	112

Quantification of muscle patterning errors from third-instar larvae stained with TxRed-conjugated phalloidin. Percents are the number of errors observed/total hemisegments scored

*n*>8 animals for all genotypes.

## Discussion

We have identified an array of consequences of the loss of *importin-α2* in *Drosophila* nerve and muscle. Rather than preventing all nuclear import and causing cell lethality, *importin-α2* mutants display specific defects in active zone development, central axon projections, and muscle patterning. These defects arise in the context of larvae whose overall patterning and development appear to be normal.

Previous work suggested roles for *Drosophila* Importin-α2 in the nervous system based on transcript expression [21]. In this study, we observe abundant expression of Importin-α2 protein in a subset of larval neurons (Figure 1). Lower levels of expression of Importin-α2 are likely to be present elsewhere in the nervous system; the location of the intensely labeled neurons of the ventral nerve cord do not correspond to the motoneurons innervating muscles 6 and 7 where altered active zone density was observed in the mutants. Although this phenotype might have arisen from a neurohumoral influence on synapse development, low levels of Importin-α2 in the motoneurons may be responsible for it in a cell autonomous fashion.

Neuronal functions of the Importin-α family have been reported recently in several systems [9], [10], [11], [13], and like the *importin-α2* phenotypes described here, can entail regulation of the synapse. In particular, studies of *Aplysia* and hippocampal neurons indicated an essential role of an Importin-α in long-term synaptic plasticity [9], [10], [48]. The phenotype at the *Drosophila* NMJ indicated a role in controlling the density of release sites and their accompanying postsynaptic receptor clusters. While there were no obvious phenotypes involving synaptic morphology and bouton number at the neuromuscular junction (Figure 2), active zone number increased by approximately 40% over wild-type (Figure 3). The additional active zones were accompanied by synaptic vesicle markers and arose in boutons with normal size and morphology. A number of molecules have been shown to decrease active zone density and disrupt receptor apposition [40], [41], [42], but they have not yet indicated a pathway involving nuclear import. Therefore, it remains unclear how active zone density is controlled by Importin-α2. Because the phenotype could be rescued by neuronal expression of Importin-α2, a reasonable hypothesis is that some unknown cargo must be imported to limit active zone density at the synaptic terminal. The density of glutamate receptor clusters in the muscle was also restored by neuronal expression of Importin-α2; the distribution of the receptors is therefore an indirect consequence of the change in presynaptic active zones.

In addition to these peripheral defects, mutants in *importin-α2* also showed defects in the organization of the central nervous system. Specifically, the axon tracts that cross the midline of the CNS and express the cell adhesion molecule Fasciclin III [49] were frequently interrupted (Figure 5). Our analysis detected only major changes in the formation of these projections; if individual axons failed to cross the midline, they would not have been counted and thus the phenotype may be more severe than our analysis revealed. The exact identity of these axons is unknown, but they may be the motoneurons RP1, 3, 4, and 5, as these are known to express Fas III [32], [49]. Defects in crossing motor axons can arise from a number of sources including axon guidance receptors [50], [51], [52], receptor tyrosine phosphatase activity [53] and cytoskeletal modulation [54], [55]. These pathways may require communication between the crossing axon and the cell nucleus to regulate gene transcription in a manner that favors axon outgrowth and enables the cell to properly respond to repulsive and attractive guidance cues. In addition, proper guidance through the commissure requires interactions with signals at the midline [56]. In this context, it is interesting that a subset of midline neurons were highly immunoreactive for Importin-α2. Thus the defect may not arise autonomously in the crossing axons but rather in signaling pathways in the midline neurons.

The stereotypical patterning and unique identification of each muscle in *Drosophila* larval body walls [47] further allowed us to recognize multiple cases of incorrect patterning in the *importin-α2* mutants. The importance of signaling pathways in establishing the normal pattern has been demonstrated previously by mutations in the *toll/dorsal* pathway and in *dystroglycan* and *POMT1.* The phenotypes of these mutants and others entail muscle duplications, absent muscles, branched muscles and errors in muscle insertion [57], [58], [59], [60], [61], [62]. All of these defects were observed in *importin-α2* mutants and arose from the loss of Importin-α2 expression in the muscle (Figure 6 and Table 1). While the mechanisms behind these patterning errors are unclear, Toll signaling at the plasma membrane is known to bring about changes in nuclear transcription [63]. It is also possible that Importin-α2 is involved in maintaining muscle integrity; mutations in *dystroglycan* and *POMT1* that affect larval muscle integrity also display patterning defects akin to and at similar frequencies to those of the importin-α2 mutants [62].

In current models of nuclear import, Importin-α binds cargo molecules destined for nuclear entry [64]. Our findings that *Drosophila* Importin-α2 is required in multiple cell types for distinct aspects of development but is not required for general development or cell viability may reflect a high degree of cargo specificity for this Importin-α [65], [66]. In addition, the cell-type specific expression of Importin-α isoforms may contribute to the specificity of the phenotype [53], [54]. Limited redundancy among Importin-α isoforms has been reported [15], further supporting a model in which they are specialized for particular cargos. As such, loss-of function phenotypes for each Importin-α will be limited and distinct, as we have observed for *importin-α2*. The identification of those phenotypes is a critical step towards identifying the mechanisms and cargo molecules through which the importins contribute to cellular processes.

## Supporting Information

Figure S1 **Active Zone Density is Normal in** ***importin1*** **Mutants.** A C Representative confocal images of larval NMJs from muscle 4 stained with antibodies against Bruchpilot magenta, GluRIIC green and HRP blue in wildtype control animals *y,w FRT42D *. Each Brp punctum is closely apposed by a corresponding GluRIIC punctum. D F Representative confocal images of *importin1* mutants *w Df3L 1S1 * stained as above. No changes in active zone apposition are apparent. Scale bar 10 m. G J Histograms of NMJ area G, Brp density H, GluRIIC density I and the ratio of Brp to GluRIIC J at wildtype and *importin1* mutants NMJs at muscle 4. No significant changes in these parameters are evident. *n* 12 NMJs for each genotype error bars represent SEM.
TIF
