# Supplementary figures and images for: Drosophila Importin-α2 Is Involved in Synapse, Axon and Muscle Development
Source: PLoS One. 2010 Dec 6;5(12):e15223. doi: 10.1371/journal.pone.0015223 (PMC2997784; doi:10.1371/journal.pone.0015223)

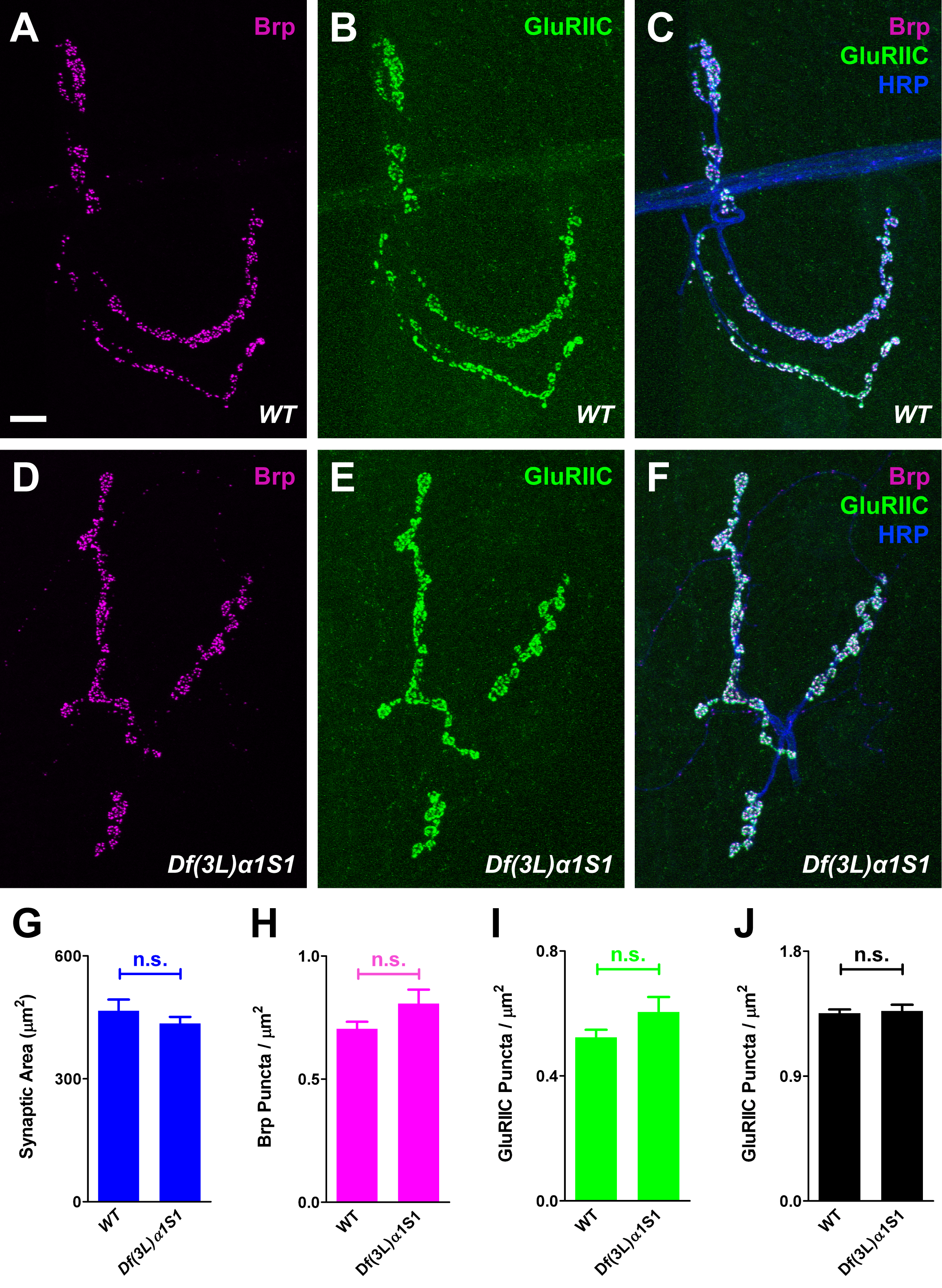

Supplement: Figure S1 — Active Zone Density is Normal in importin1 Mutants. A C Representative confocal images of larval NMJs from muscle 4 stained with antibodies against Bruchpilot magenta, GluRIIC green and HRP blue in wildtype control animals y,w FRT42D . Each Brp punctum is closely apposed by a corresponding GluRIIC punctum. D F Representative confocal images of importin1 mutants w Df3L 1S1 stained as above. No changes in active zone apposition are apparent. Scale bar 10 m. G J Histograms of NMJ area G, Brp density H, GluRIIC density I and the ratio of Brp to GluRIIC J at wildtype and importin1 mutants NMJs at muscle 4. No significant changes in these parameters are evident. n 12 NMJs for each genotype error bars represent SEM. TIF [file pone.0015223.s001.tif]
